# How the New Interacts With the Old? Hippocampal Processing During Memory Encoding of Creative Associations With Remote or Close Inherent Semantic Relatedness

**DOI:** 10.1002/hbm.70381

**Published:** 2025-10-23

**Authors:** Jingjing Yang, Zhi Zhang, Ziyi Li, Ze Zhang, Jing Luo

**Affiliations:** ^1^ Beijing Key Laboratory of Learning and Cognition, School of Psychology Capital Normal University Beijing China; ^2^ State Key Laboratory of Cognitive Neuroscience and Learning & IDG/McGovern Institute for Brain Research Beijing Normal University Beijing China; ^3^ Department of Psychology Shaoxing University Shaoxing China

**Keywords:** alternate uses task, creative association, hippocampus, semantic relatedness, subsequent memory effect

## Abstract

Creativity means the formation of novel and useful associations. Meanwhile, the role of the hippocampus in episodic memory and some forms of creative thinking has been identified, but it remains unclear how the hippocampus participates in the formation of memory for creative associations. In particular, considering creative associations are often formed on the basis of old ones, it is important to identify how the hippocampus and its associated neural network represent the interactions between the new and old associations during the encoding of creative associations. Thus, using the subsequent memory effect (SME) paradigm, the present study asked participants to learn a set of creative combinations (a common object paired with a creative alternate use, for example, basketball‐buoy, which means a *basketball* is used as a *buoy*) during fMRI scanning. Moreover, we also quantified the degree of pre‐existing semantic connections individually according to subjective ratings of inherent semantic relatedness between the objects and their alternate uses in the relatedness judgment task, resulting in a 2 (memory: remembered vs. forgotten) by 2 (semantic relatedness: remote vs. close) factorial design. Multivariate analysis revealed higher inter‐item hippocampal pattern similarity for remembered relative to forgotten trials in both close relatedness and remote relatedness conditions, indicating that hippocampal representations become less separable supporting successful memory for creative associations. However, univariate analyses of the hippocampus and its neural network showed that enhanced hippocampal activation was associated with successful encoding in the remote relatedness but not close relatedness condition, whereas increased hippocampal functional connectivity with prefrontal and parietal cortices contributed to successful memory in the close relatedness but not remote relatedness condition. These observations suggest that hippocampal‐dependent processes and distributed hippocampal network patterns selectively support successful memory for creative associations with either remote or close inherent semantic relatedness, which implies the interactions between pre‐existing semantic connections and newly formed creative associations.

## Graphical abstract

Our findings suggest that hippocampal‐dependent processes and distributed hippocampal network patterns selectively support successful memory for creative associations with either remote or close inherent semantic relatedness, which implies the interactions between pre‐existing semantic connections and newly formed creative associations.

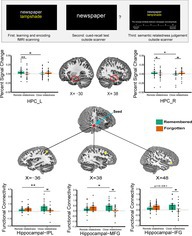

## 1 Introduction

A fundamental function of the hippocampus is to form associations among individually encoded elements (Henke et al. 1999; Staresina and Davachi 2009; Ranganath 2010) and bind them into a unified representation (Davachi 2006; Eichenbaum 2017). This prominent function is critical to episodic memory formation, where the perceptual, semantic, and affective features of events and their sequences are integrated into a coherent representation that supports flexible memory retrieval (Tulving 2002; Davachi 2006; Eichenbaum 2017). The hippocampus exhibits consistent activation across diverse memory tasks (Eichenbaum et al. 2007; Ranganath 2010), especially in the subsequent memory effect (SME) paradigm, where the level of hippocampal activity during encoding predicts subsequent successful retrieval (Brewer et al. 1998; Wagner et al. 1998; Qin et al. 2007, 2009; Kim 2011; Becker et al. 2017; Cooper and Ritchey 2020). In addition, the encoding of new information always interacts with prior knowledge (Poppenk et al. 2010; Zeithamova, Schlichting, and Preston 2012; Preston and Eichenbaum 2013; Schlichting et al. 2015; Schlichting and Preston 2015, 2016; Liu et al. 2017). More specifically, the hippocampus and its network, particularly its functional interactions with the prefrontal cortex and other neocortical regions, have been shown to play distinct roles in supporting the memory encoding of new information that is incongruent or congruent with pre‐existing knowledge or schemas. For the hippocampus, researchers have proposed that it is more sensitive to the novel information incongruent with existing schemas (van Kesteren et al. 2012; Preston and Eichenbaum 2013). Empirical data demonstrated that hippocampal engagement was positively predictive of successful encoding of incongruent information (van Kesteren et al. 2013, 2014; Reggev et al. 2016). However, hippocampal involvement is not invariably predictive of successful memory formation. Enhanced hippocampal activation was also found to be associated with false memories, which likely result from increased interference between overlapping information acquired across multiple learning experiences (van Kesteren et al. 2012; St. Jacques et al. 2013; Schlichting and Preston 2015; Vijayarajah and Schlichting 2023), suggesting potential negative consequences of enhanced hippocampal involvement in resisting interference from the encoding context.

For the hippocampal‐neocortical connectivity, however, previous studies have shown that enhanced functional connectivity between the hippocampus and neocortical regions, especially the prefrontal cortex, predicts successful memory for new events relevant to prior knowledge or schemas (Poppenk et al. 2010; van Kesteren et al. 2013, 2020), which is believed to reflect the integration of new information into pre‐existing knowledge (Preston and Eichenbaum 2013; Schlichting and Preston 2016; Sommer 2017). For instance, studies found that greater functional connectivity between the prefrontal cortex and hippocampus predicted memory for schema‐related information (Liu et al. 2017, 2018). Therefore, the hippocampus and its associated network may differentially contribute to the encoding of new information, modulated by the congruency between new information and pre‐existing knowledge. While the hippocampus preferentially supports the associative processing of highly novel information, the hippocampal network facilitates the integrative processing of new events with existing knowledge.

An intriguing question is the specific role of the hippocampus and its associated network in forming creative associations (Zhang et al. 2020; Benedek et al. 2020, 2023). Creative thought is fundamentally concerned with establishing novel and useful associations (i.e., creative associations) among seemingly unrelated elements or concepts (Barron 1955; Mednick 1962; Ward 2008; Runco and Jaeger 2012; Beaty et al. 2017; Benedek and Fink 2019), a process closely related to the core function of the hippocampus. Previous studies have shown that patients with hippocampal damage exhibit deficits in imagination, creative thinking, and insight problem solving (Hassabis et al. 2007; Duff et al. 2013). Converging neuroimaging evidence further highlights the involvement of the hippocampus in a range of creative cognition tasks, including the alternate uses task (Beaty et al. 2018; Madore et al. 2019), insight problem solving (Luo and Niki 2003), creative cognitive reappraisal (Wu et al. 2019), the formation of strengthened creative associations (Benedek et al. 2020), and the processing of creative designs (Ren et al. 2020, 2023). As a key neural mechanism underlying both episodic memory and creative thought, the hippocampus has been found to be recruited in divergent thinking and imagining the future based on past experiences (Madore et al. 2016, 2019; Cabeza et al. 2020; Thakral et al. 2020), suggesting the role of the hippocampus in binding mnemonic or episodic elements into holistic mental representations, including imaginary or creative ones. Further studies have demonstrated the specific contribution of the hippocampus to integrating the two essential features of creativity—novelty and usefulness—during the processing of insightful chunk decomposition (Huang et al. 2015) and constraint relaxation (Huang et al. 2018), as well as the mental representations of creative designs (Ren et al. 2020, 2023) and metaphorical solutions (Yu et al. 2021).

In addition to the hippocampus, the hippocampal (or medial temporal lobe) network was also involved in various forms of creative cognition (Huang et al. 2016; Ren et al. 2023). For example, the parahippocampal gyrus was found to differentially mediate the mental representations of “incremental” and “radical” product designs by enhancing its functional connectivity with nodes in the dorsal spatial‐motor pathway and the ventral conceptual pathway, respectively (Huang et al. 2016). The parahippocampal gyrus also distinguished truly creative designs (i.e., novel and useful, NU design) from designs that were only novel but not useful (NS design) through increasing its functional connectivity with the hippocampus during NU design processing, and with regions related to inhibitory control (right superior frontal gyrus and left medial frontal gyrus) and emotional arousal (amygdala) during NS design processing (Ren et al. 2023). Despite these advancements, we still know little about how the hippocampus and its neural network support the memory encoding of new creative associations.

A recent hypothesis regarding the neurocognitive basis of “insight memory advantage” (IMA)—the phenomenon whereby insight solutions accompanied by Aha! experiences are better remembered later—proposes that IMA arises from insight‐induced prediction errors, which could be detected by the hippocampus (Becker and Cabeza 2025). In support of the hypothesis, a neuroimaging study on visual insight problems (identifying the difficult‐to‐recognize images of real‐world objects) revealed hippocampal involvement and its functional connectivity with visual regions during insight. Furthermore, hippocampal engagement was positively associated with subsequent memory (Becker et al. 2025). The prediction error hypothesis underlying IMA may also apply to other forms of creative thinking. However, even when creativity level (and hence prediction error) in forming creative associations is comparable, the hippocampus and its neural network may contribute differentially to memory encoding, depending on the pre‐existing semantic connections between the to‐be‐associated elements. This constitutes the central focus of the current study.

The present study investigated hippocampal activity patterns in the successful encoding of creative associations with relatively remote or close pre‐existing semantic connections to elucidate how the hippocampus represents the possible interactions between newly formed creative associations and existing semantic connections. Although creative associations (i.e., novel and useful associations) are usually supposed to be formed between the unrelated concepts, for instance, the alternate use (AU) of a *newspaper* as a *lampshade* forms new associations between the two seemingly unrelated concepts, *newspaper* and *lampshade*, these concepts (i.e., *newspaper* and *lampshade*) may not be entirely unrelated based on pre‐existing semantic knowledge. Rather, there is still a degree of existing semantic connections (i.e., old associations) among these elements. In the present study, we estimated the degree of pre‐existing semantic connections by asking participants to subjectively rate the inherent conceptual relatedness or semantic similarity between the target objects and their creative AU ideas (e.g., the inherent conceptual or semantic relatedness between *newspaper* and *lampshade*) for each object‐AU creative combination. Using the SME paradigm, we then examined how the remembering or forgetting of the encoded creative combinations, as well as the subjectively rated remote or close semantic connections influenced the involvement of the hippocampus and its neural network during the encoding. That is, participants first learned 100 creative object‐AU combinations, then completed a cued‐recall test in which each target object served as a cue for retrieving its paired AU idea, followed by rating the inherent semantic relatedness between the elements (i.e., the objects and their AU ideas). To ensure that memory encoding of creative associations was not confounded by any prior exposures and thereby enhance the reliability of neuroimaging data, the judgment of inherent semantic relatedness was administered at the end of the experimental procedures, after both the encoding phase and cued‐recall test. However, it should be noted that prior experiences of learning and retrieving creative associations might alter the ratings of semantic relatedness between the two concepts and lead it to be less accurate. Thus, an additional behavioral control experiment was conducted to rule out the possibility that prior learning and memory retrieval experiences altered semantic relatedness judgments.

In the present study, we focused on the dissociable roles of the hippocampus and its neural network in encoding creative associations into memory, which may be modulated by the pre‐existing semantic connections between the to‐be‐combined elements or concepts. Specifically, based on the theoretical and empirical evidence in previous studies (van Kesteren et al. 2012; Preston and Eichenbaum 2013; Reggev et al. 2016), successful association formation (i.e., subsequent remembering of associations) between two remotely related concepts might rely on the associative processing mediated by the hippocampus, which contributes to the binding of separate elements into a unified mental representation (Davachi 2006; Qin et al. 2007, 2009; Kim 2011; Eichenbaum 2017; Cooper and Ritchey 2020). That is, increased activation of the hippocampus during encoding would be associated with successful memory retrieval of creative combinations under the conditions where the semantic relatedness between the target object and creative AU idea is relatively remote.

However, when the inherent semantic relatedness between two elements is relatively close, excessive hippocampal binding in the encoding of creative combinations might be less beneficial, or even detrimental, for subsequent retrieval, as it could heighten interference between the new and old associations (van Kesteren et al. 2012; St. Jacques et al. 2013; Schlichting and Preston 2015). Under these conditions, increased functional connectivity between the hippocampus and other cortical regions (i.e., the hippocampal network) would be more advantageous, given that the hippocampal network (hippocampal–prefrontal interactions in particular) could specifically contribute to the integration of new associations with pre‐existing schemas to enhance memory retention (Preston and Eichenbaum 2013; Schlichting and Preston 2016; Sommer 2017; Liu et al. 2017, 2018). Therefore, the present study hypothesized that there would be a potential functional dissociation between the hippocampal region and hippocampal network in the encoding of creative combinations with varying degrees of semantic relation. The independent hippocampal encoding facilitates memory formation for the creative combinations of relatively remotely related concepts, while the hippocampal network supports memory for the creative combinations of relatively closely related concepts.

## 2 Methods

### 2.1 Participants

Thirty‐four university students (15 males and 19 females, mean age ± SD, 23.06 ± 2.17) took part in this study. All participants were right‐handed with normal or corrected‐to‐normal vision, and reported no history of neurological disorders. All participants signed written informed consent forms before the experiment and were paid after participation. And all the experimental protocols were approved by the ethics committee of the Center for Biomedical Imaging Research, Tsinghua University. Another independent cohort of 23 healthy participants (13 males, mean age ± SD, 22.00 ± 1.86) were recruited for a behavioral control experiment using the same paradigm to validate the effectiveness of semantic relatedness judgments.

### 2.2 Materials

**FIGURE 1:**
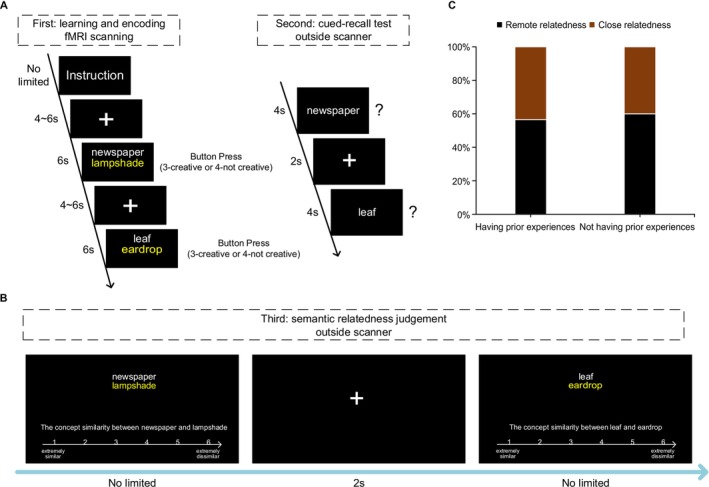
(A, B) Procedures of the experiment, and the experiment consisted of three phases. During the encoding phase, in each run, the instruction was first presented on the screen, and participants were instructed to learn and indicate with a button press whether the described use for this object was creative or not (3‐creative; 4‐not creative). They were asked to press a button using the index or middle finger of the right hand. Then during the post‐scan testing phase, participants first completed a surprise cued‐recall memory test, followed by a semantic relatedness judgment task. (C) Ratings of semantic relatedness between the objects and their alternate uses were not influenced by prior experiences of encoding and retrieval in the supplemental behavioral experiment.

The formal experiment consisted of 100 ordinary objects, each paired with one alternate use (object‐AU combination). In each combination, the creative association was formed between the common object and its alternate use, and no objects or alternate uses were repeated across all object‐AU combinations. Based on an independent group of 10 participants using a 5‐point Likert scale, the average creativity of these combinations was 3.43 (SD = 0.27), with an average novelty score of 3.85 (SD = 0.33), usefulness score of 3.51 (SD = 0.31), and understandability score of 4.53 (SD = 0.28). Additionally, 20 different common objects paired with their non‐creative usages (conventional or novel but useless) served as filler stimuli without contributing to the primary dataset. All stimuli were presented textually, with objects at the top and their corresponding alternate uses below (e.g., newspaper‐lampshade, depicted in Figure 1A).

### 2.3 Procedures

The whole experiment consisted of three phases: the learning and encoding task in the MRI scanner, the cued‐recall memory test, and the semantic relatedness judgment task outside the scanner. During the fMRI scanning phase, participants were instructed to learn 120 pairs in the learning and encoding task. Following that, participants were asked to complete two post‐scan tasks. First, participants performed a surprise cued‐recall memory test for object‐AU creative combinations, in which objects from the learning phase were presented again as cues, and participants were asked to recall the corresponding alternate use for each object. Second, in the semantic relatedness judgment task, they were required to assess the inherent semantic relatedness between the object and its alternate use for each object‐AU combination.

#### 2.3.1 Learning and Encoding Task

Participants initially completed the learning and encoding task during fMRI scanning. The experiment comprised a total of 120 trials divided into four runs with 30 trials each run, of which 25 object‐AU combinations and 5 filler stimuli. The order of trials within each run was randomized, and the sequence of the four runs was counterbalanced across participants. For each trial, the participants were shown an object and its use for 6 s. During this period, they were instructed to evaluate whether the presented use for this object was creative or not by pressing a button with their right hand within the presentation. Participants were expected to concentrate on comprehending the stimulus for the entire display period. An inter‐trial interval with a fixation cross for 4 to 6 s was presented between two trials (Figure 1A).

#### 2.3.2 Cued‐Recall Memory Test

Upon completion of the encoding phase with the MRI scanning, participants entered another quiet room and performed two post‐scan tests to validate memory performance and semantic relatedness. First, memory for object–AU combinations was assessed by an unexpected cued‐recall test (participants did not know in advance they would be given such a memory test during the encoding task). In the test, the 100 objects from the previously encoded object–AU combinations were presented in a random order. Participants were required to recall the associated alternate use for each object (e.g., recalling “lampshade” when cued with “newspaper”). Each object appeared on the screen for 4 s, during which the participant recalled the alternate use (illustrated in Figure 1A). If they remembered, they pressed the F key rapidly and input the answer. If they did not remember, the next trial would be presented after a maximum time of 4 s. The answer was scored as correct (i.e., remembered) only if it was the identical term as learned or reflected the same alternate use despite minor variations that did not alter the original meaning of the idea (it was determined by two experienced raters). Incorrect answers or no response were treated as forgotten.

#### 2.3.3 Semantic Relatedness Judgment Task

After the cued‐recall test, participants performed the relatedness judgment task, in which they were presented with the same 100 object‐AU combinations in a random order (Figure 1B). They were required to rate the inherent semantic relatedness between each object and its AU idea (the conceptual similarity between the object concept and the alternate use) on a 6‐point scale, with “1” indicating extremely similar and “6” indicating extremely dissimilar. Thus, higher scores indicated more remote semantic connections between the object and their associated alternate uses. In the subsequent statistical analysis, median splits were performed for each participant's ratings of the object‐AU combinations. Before the beginning of each task, task‐related instructions were explained, and participants performed practice trials to become familiar with the experimental procedure.

### 2.4 Behavioral Control Experimental Procedures

In the behavioral control experiment, 30 object‐AU combinations were selected from the fMRI experimental materials and divided into two equal groups (Group A and Group B), with 15 stimuli in each. For half of the participants, the learning and cued‐recall test were first performed with the Group A combinations, followed by a 6‐point rating of the inherent semantic relatedness for each combination in both Groups A and B. For the other half, participants first completed the encoding and retrieval of creative combinations in Group B, followed by a rating of semantic relatedness for all combinations in Groups A and B. The assignment of Groups A and B was counterbalanced across all participants.

### 2.5 MRI Acquisition

The fMRI scanning was performed on a 3T Philips Achieva 3.0T TX MRI scanner with a 32‐channel head coil at the Center for Biomedical Imaging Research, Tsinghua University. Functional images were acquired using a T2*‐weighted echo‐planar imaging (EPI) sequence based on blood oxygenation level dependent (BOLD) contrast. The following acquisition parameters were used: TR = 2000 ms, TE = 30 ms, FOV = 200 mm × 200 mm, FA = 90°, 64 × 63 matrix, 30 slices, voxel size = 3.13 mm × 3.13 mm × 3.0 mm. High‐resolution structural T1‐weighted brain images were acquired for each participant (TR = 7.56 ms, TE = 3.70 ms, FOV = 256 mm × 256 mm, FA = 8°, 160 slices, voxel size = 1.0 mm × 1.0 mm × 2.0 mm).

### 2.6 Imaging Preprocessing

The imaging data were analyzed using SPM12 software (Statistical Parametric Mapping, Welcome Department of Cognitive Neurology, London, UK) implemented within MATLAB 2018a. For preprocessing, the images for each subject were corrected for slice acquisition timing and realigned for head motion correction. Then the images were spatially normalized to a standard EPI template within SPM, and smoothed with a 6‐mm full‐width at half‐maximum Gaussian kernel.

### 2.7 Univariate GLM Analysis of Hippocampal Activation

For subsequent statistical analysis, first, according to the post‐scan cued‐recall test, the object‐AU combinations were classified into remembered condition and forgotten condition for each participant. If the AU idea of the object was correctly recalled, the corresponding object‐AU combination (from the fMRI scan) was classified in the remembered condition; if failed or incorrect, the corresponding object‐AU combination was classified in the forgotten condition. Then, to conduct a contrast of remote versus close semantic connections, we classified the semantic relatedness between the objects and their alternate uses into two different conditions. Based on each participant's relatedness ratings in the relatedness judgment task, for each object‐AU combination, if the inherent relatedness between the object and its alternate use is strong, that is, the conceptual similarity score between the alternate use and the object is equal to or less than 3, the corresponding object‐AU combination is classified in the “close” condition; while if the inherent relatedness between the object and its alternate use is weak, that is, the conceptual similarity score between the elements is equal to or larger than 4, the corresponding object‐AU pair is classified in the “remote” condition. Thus, this resulted in a 2‐by‐2 full factorial design (i.e., semantic relatedness: remote versus close, memory: remembered versus forgotten). Consequently, there were four conditions: (1) remote and remembered: object‐AU combinations with remote semantic relatedness that were later remembered; (2) remote and forgotten: combinations with remote semantic relatedness that were later forgotten; (3) close and remembered: combinations with close semantic relatedness that were later remembered; (4) close and forgotten: combinations with close semantic relatedness that were later forgotten. Furthermore, based solely on each participant's behavioral performance in the two post‐scan tasks, we excluded those participants whose trial numbers in certain conditions were fewer than eight (data from 14 participants were excluded). The data of the remaining 20 participants were included in the formal statistical analysis. That is, for these participants, the number of remembered and forgotten items reached at least eight in both the remote and close relatedness conditions.

The effects were estimated using the General Linear Model (GLM). At the first‐level analysis, four regressors of interest were defined as the trials in each of the conditions: Remote_Remembered, Remote_Forgotten, Close_Remembered, and Close_Forgotten. Four regressors of interest were modeled and convolved with the canonical hemodynamic response function (HRF). Then, an additional regressor was modeled for filler trials that were out of interest, and six motion parameters were also included to control for head movement‐related variability. Linear contrasts were used to obtain specific condition effects for each participant.

For the second‐level analysis, the resulting contrast parameter estimate images from each participant were submitted to a random effects model, using a 2 × 2 full factorial analysis of variance (ANOVA) with the factors of semantic relatedness (remote, close) and memory (remembered, forgotten) to examine the main effects of semantic relatedness and memory, as well as the interaction between these two factors. For the whole‐brain analyses, a voxelwise threshold of *p* < 0.001 (uncorrected) was used, and 10 or more contiguous voxels were reported. Moreover, a relatively lenient threshold of *p* < 0.005 (uncorrected) was also used in the interaction analysis to detect activation in the hippocampus.

The bilateral hippocampus was chosen as the region of interest (ROI), which was defined by superimposing the activated clusters on the structurally defined templates. The activated clusters were derived from the group‐level interaction analysis using a threshold of uncorrected *p* < 0.005. The structural templates were further defined using the automated anatomical labeling (AAL) templates from the WFU Pick Atlas toolbox (Version 3.0, http://fmri.wfubmc.edu/software/PickAtlas). The percentage signal changes within the ROI were extracted for each condition separately for each participant using MarsBar (http://marsbar.sourceforge.net/).

### 2.8 Task‐Dependent Functional Connectivity Analysis of Hippocampal Network

We examined hippocampus‐based functional connectivity changes via a generalized form of task‐dependent psychophysiological interaction (gPPI) analysis. The hippocampal seed was separately defined as a 6‐mm sphere centered at the local peak of corresponding clusters showing significant interaction effects between semantic relatedness and memory in the univariate GLM analysis. The physiological activity of a given seed region was computed as the mean time series of all voxels, and then was deconvolved to estimate neural activity. Next, four PPI regressors were obtained by multiplying the estimated neural activity from the seed region with a vector coding for effects of each condition, forming four PPI vectors at the individual‐subject level. They were further convolved with a canonical HRF to form four PPI regressors of interest. Contrast images corresponding to PPI effects from the individual level were then entered into a 2 (semantic relatedness) × 2 (memory) ANOVA on the second‐level group analysis. Significant clusters were determined using the threshold of uncorrected *p* < 0.001 (cluster size > 10).

### 2.9 Prediction Analysis of Hippocampal‐ Neocortical Connectivity With Memory Performance

To investigate the relationship of hippocampal functional connectivity and memory performance, significant clusters in the neocortical regions showing interaction effects between semantic relatedness and memory in the gPPI analysis were defined as the ROIs and further constrained by respective structural templates from the WFU PickAtlas. Then mean *t* values were extracted from resultant contrast images in each ROI. We employed a machine‐learning approach with balanced fourfold cross‐validation to examine whether greater hippocampal coupling with these regions during learning could predict better memory. We entered cued‐recall accuracy for each individual as the dependent variable and hippocampal functional connectivity with these regions as the independent variable. Then, we estimated *r*_(predicted, observed)_ to measure how well the independent variable predicts the dependent variable using a balanced fourfold cross‐validation procedure. Data were divided into four folds. A linear regression model was built using three folds to predict the remaining data in the left‐out one fold, and the procedure was repeated four times. Then, a final *r*_(predicted, observed)_ was recorded as the average of four repetitions. Finally, a nonparametric testing approach was used to test the statistical significance of the model by generating 1000 surrogate data sets under the null hypothesis of *r*_(predicted, observed)_. The statistical significance (*p* value) of the model was determined by measuring the percentage of generated surrogate data greater than the *r*_(predicted, observed)_.

### 2.10 ROI‐Based Pattern Similarity Analysis of Hippocampal Representation

We determined the left and right entire hippocampus as ROIs using the AAL atlas. To assess multivoxel pattern similarity associated with the encoding of creative associations, we modeled each item with a duration of 6 s as a separate regressor, convolved with a canonical HRF in SPM12, resulting in a total of 120 regressors. Then contrast images for each item versus fixation, generated at the individual level analysis, were submitted to subsequent inter‐item pattern similarity analysis for the hippocampal ROIs. Specifically, for the four conditions, we extracted voxel‐wise brain activation estimates for each item within the same condition from the hippocampal ROIs. Pairwise correlations were computed between each two of the items in each condition among distributed voxels of each ROI (Figure 6A). The correlation coefficients were transformed into Fisher's *z*‐scores and then averaged to obtain the similarity scores, separately for each participant. Then the resultant similarity values were submitted to a 2 (semantic relatedness) × 2 (memory) repeated‐measures ANOVA for the group‐level analysis to investigate differences in pattern similarity between remembered and forgotten trials in the “remote” and “close” conditions.

## 3 Results

### 3.1 The Validity of Semantic Relatedness Judgment

In order to examine whether prior experiences of learning and retrieval affected the subsequent judgments of semantic relatedness, a chi‐square test was performed on the data from the supplemental control experiment. The result revealed that the proportion of remote and close items was not influenced by prior experiences of learning and retrieval (χ^2^ = 0.858, *p* = 0.354, Figure 1C). These indicate that participants were able to assess semantic relatedness independently of their prior learning and retrieval experiences.

### 3.2 Behavioral Results of the fMRI Experiment

After excluding the participants whose trial numbers were fewer than eight in certain conditions, out of the 100 object–AU combinations, for the remaining 20 participants, there were an average of 24.6 (SD = 11.73), 28.75 (SD = 12.53), 22.9 (SD = 12.43), and 23.75 (SD = 10.81) trials for the Remote_Remembered, Remote_Forgotten, Close_Remembered, and Close_Forgotten conditions, respectively. Moreover, participants were asked to evaluate the creativity of the 100 object–AU combinations during the encoding phase in the scanner. By examining the creativity evaluation, we found that there were an average of 62.6% combinations (SD = 18.75%) being evaluated as creative.

**FIGURE 2:**
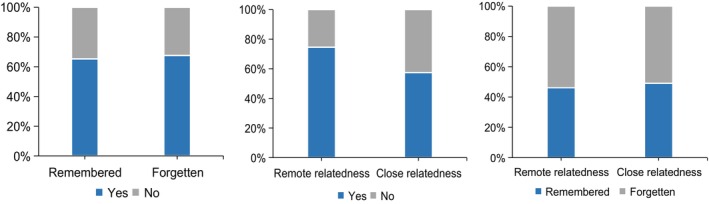
The results of behavioral response in fMRI experiment (left: phase 1 vs. phase 2; middle: phase 1 vs. phase 3; right: phase 2 vs. phase 3). “Yes” indicated participants evaluated the object‐AU combinations as creative during the encoding phase in fMRI scanning; “No” indicated participants evaluated the object‐AU combinations as non‐creative during the encoding phase.

In addition, to examine the evaluated creativity for remembered and forgotten items, a chi‐square test was performed on the behavioral data of phase 1 (encoding) and phase 2 (cued‐recall test); the result showed that there was no difference for the creativity evaluation of remembered and forgotten items during encoding (χ^2^ = 1.102, *p* = 0.294). Another chi‐square test was also performed on the behavioral data of phase 1 (encoding) and phase 3 (semantic relatedness judgment); there was a significant difference for the creativity evaluation of remote relatedness items and close relatedness items (χ^2^ = 61.872, *p* < 0.001). Participants tended to evaluate object‐AU combinations as creative in the “remote” condition. Similarly, a chi‐square test comparing the behavioral data from phase 2 and phase 3 revealed no significant difference for the proportion of remembered items between the “remote” and “close” conditions (χ^2^ = 1.771, *p* = 0.183) (Figure 2). Besides, a 2 (memory: remembered vs. forgotten) by 2 (semantic relatedness: remote vs. close) repeated‐measures ANOVA for the proportion of object‐AU combinations evaluated as creative revealed a significant main effect of relatedness (*F*_(1, 19)_ = 8.44, *p* = 0.009); the proportion of creative items in the remote relatedness condition was higher than in the close relatedness condition, and neither the main effect of memory nor the interaction effect was significant. That is, in both the close and remote relatedness conditions, there was no significant difference for the proportion of creative items between the remembered and forgotten conditions.

### 3.3 Imaging Results

#### 3.3.1 Whole‐Brain Analysis Results

##### 3.3.1.1 Main Effect of Memory

Neural activity was examined by comparing the remembered and forgotten conditions (remembered vs. forgotten). This analysis showed increased neural activity in the bilateral inferior frontal gyrus, left superior frontal gyrus, medial frontal gyrus, and precentral gyrus. Decreased neural activity was identified in the left posterior cingulate cortex, right middle temporal gyrus, superior temporal gyrus, right precuneus, and inferior parietal lobule (Table 1 and Figure S1).

**TABLE 1:** Brain regions associated with the main effect of memory.

Brain regions	Hemisphere	Brodmann's area	MNI coordinates			*T*	*K*
			*x*	*y*	*z*		
Remembered > Forgotten							
Superior Frontal Gyrus	Left	8	−12	18	56	4.07	141
Superior Frontal Gyrus	Left	8	−8	38	50	3.82	13
Medial Frontal Gyrus	Left	6	−4	10	58	3.42	10
Inferior Frontal Gyrus	Right	46	44	30	10	4.78	90
Inferior Frontal Gyrus	Left	46	−42	42	−2	5.84	656
Precentral Gyrus	Left	6	−38	−4	32	4.41	270
Forgotten > Remembered							
Middle Temporal Gyrus	Right	21	66	−12	−14	4.73	32
Superior Temporal Gyrus	Right	22	64	−30	10	3.81	66
Inferior Parietal Lobule	Right	40	46	−54	42	3.60	12
Inferior Parietal Lobule	Right	40	62	−46	20	4.66	261
Supramarginal Gyrus	Right	40	58	−42	30	3.97	
Precuneus	Right	7	18	−60	22	5.82	2338
Cuneus	Left	18	−16	−66	22	5.29	
Posterior Cingulate	Left	23	−6	−44	22	3.82	20

*Note:* Threshold of voxel levels: *T* = 3.20, *p* < 0.001(uncorrected), *k* = 10.

##### 3.3.1.2 Main Effect of Semantic Relatedness

Neural activity was examined by comparing the “remote” and “close” conditions (remote vs. close). This analysis revealed increased neural activity in the bilateral hippocampus, bilateral postcentral gyrus, and left superior temporal gyrus. Decreased neural activity was observed in the right middle frontal gyrus, superior frontal gyrus, and medial frontal gyrus (Table 2 and Figure S2).

**TABLE 2:** Brain regions associated with the main effect of semantic relatedness.

Brain regions	Hemisphere	Brodmann's area	MNI coordinates			*T*	*K*
			*x*	*y*	*z*		
Remote > Close							
Hippocampus	Right		28	−8	−16	4.40	30
Hippocampus	Left		−28	−20	−16	4.04	14
Superior Temporal Gyrus	Left	22	−52	−4	4	3.61	12
Postcentral Gyrus	Right	4	54	−10	18	3.76	78
Postcentral Gyrus	Left	4	−56	−4	24	3.76	39
Close > Remote							
Medial Frontal Gyrus	Right	6	10	22	48	3.96	19
Middle Frontal Gyrus	Right	45	44	26	30	3.56	23
Superior Frontal Gyrus	Right	8	4	26	54	3.56	15

*Note:* Threshold of voxel levels: *T* = 3.20, *p* < 0.001(uncorrected), *k* = 10.

##### 3.3.1.3 Interaction Effect

**FIGURE 3:**
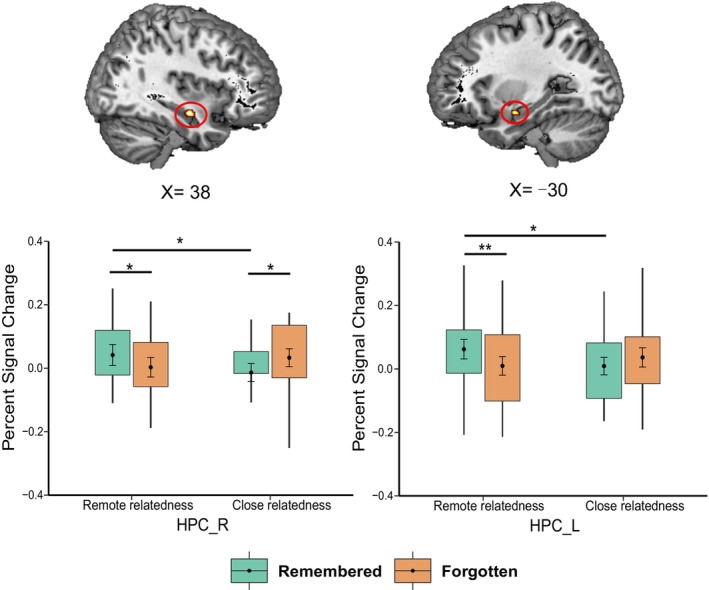
Significant clusters in the left hippocampus (MNI, peak at −30, −6, and −16) and right hippocampus (MNI, peak at 38, −10, and −16) showing interaction effects between semantic relatedness and memory. Bar graphs show that the percent signal changes in the left and right hippocampal ROIs, which were obtained by superimposing anatomically defined masks on the functionally activated clusters in the interaction effects. *p < 0.05; **p < 0.01; MNI, Montreal Neurological Institute; HPC, hippocampus; R, right; L, left.

To investigate the neural mechanisms underlying the interaction between semantic relatedness and memory, we contrasted remembered items with forgotten items in the remote versus close relatedness condition. This analysis revealed significant clusters in the bilateral hippocampus (Table 3). To further detect differences among these four conditions, we performed follow‐up ROI analyses, which revealed higher activation in the bilateral hippocampus for remembered relative to forgotten trials in the “remote” condition (*t*_(19)HPC_R_ = 2.38, *p* = 0.028; *t*_(19)HPC_L_ = 3.24, *p* = 0.004). However, in the “close” condition, we observed significant decreased activation in the right hippocampus for the remembered relative to forgotten trials (*t*_(19)HPC_R_ = −2.58, *p* = 0.018), and a similar but non‐significant trend in the left hippocampus (*t*_(19)HPC_L_ = −1.50, *p* = 0.149; Figure 3).

**TABLE 3:** Brain regions associated with the interaction between semantic relatedness and memory.

Brain regions	Hemisphere	Brodmann's area	MNI Coordinates			*T*	*K*
			*x*	*y*	*z*		
Interaction effect							
Hippocampus*	Right		38	−10	−16	3.20	16
Hippocampus*	Left		−30	−6	−16	3.24	8

*Note:* Threshold of voxel levels: *T* = 3.20, *p* < 0.001(uncorrected), *k* = 10; *indicated *p* < 0.005 (uncorrected).

#### 3.3.2 PPI Results

**FIGURE 4:**
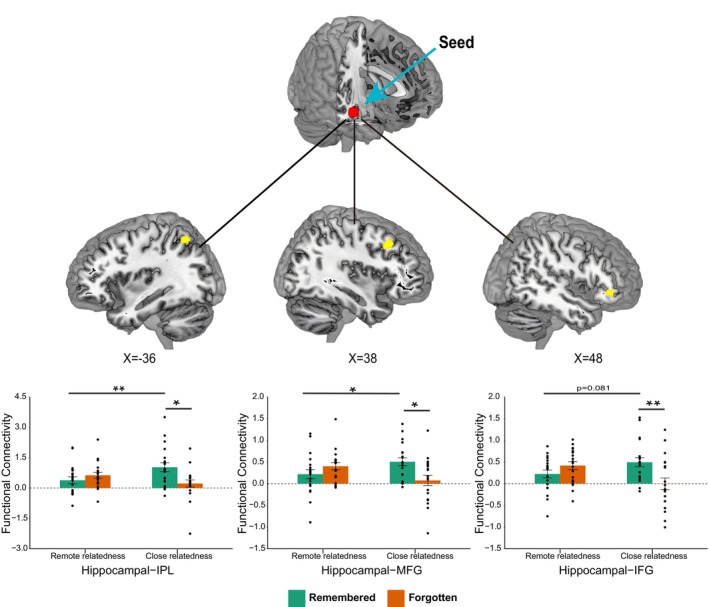
Distinct hippocampal functional connectivity in the encoding of creative associations. Significant clusters in the right IFG, MFG, and left IPL show interaction effects between semantic relatedness and memory in gPPI functional connectivity analysis. Bar graphs represent hippocampal connectivity with these regions for remembered items and forgotten items in the remote and close relatedness conditions. *p < 0.05; **p < 0.01; IFG, inferior frontal gyrus; MFG, middle frontal gyrus; IPL, inferior parietal lobule.

**FIGURE 5:**
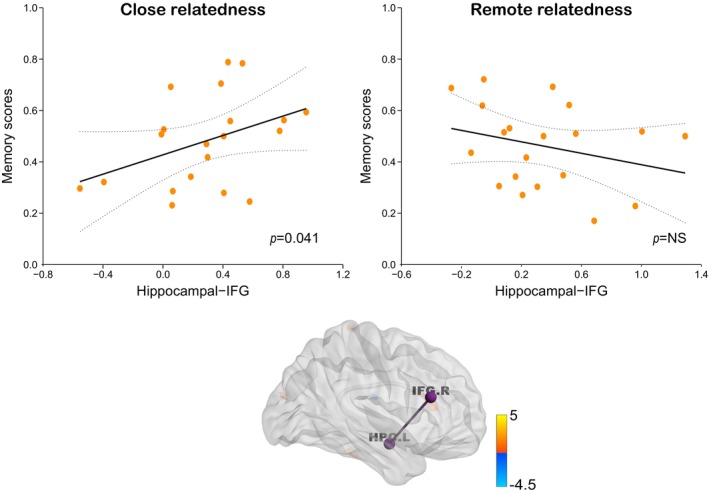
Functional connectivity between the left hippocampus and right IFG was positively predictive of better memory performance in the close condition, but not in the remote condition. Dotted lines indicate 95% confidence intervals and solid line indicates the best linear fit. IFG, inferior frontal gyrus; HPC, hippocampus; R, right; L, left.

To investigate how pre‐existing semantic connections affect the hippocampal functional connectivity in memory for creative association, we conducted a gPPI analysis to identify functional coupling of the hippocampus with every other voxel of the brain. This analysis revealed significant interaction effects in the middle frontal gyrus, inferior frontal gyrus, and inferior parietal lobule (Table S1). Further analysis revealed significantly higher hippocampal functional coupling with these regions for the remembered relative to forgotten items in the “close” condition (*t*_(19)MFG_R_ = 2.80, *p* = 0.011; *t*_(19)IFG_R_ = 2.89, *p* = 0.009; *t*_(19)IPL_L_ = 2.65, *p* = 0.016), but not in the “remote” condition (both *t*_(19)_ < 1.74, *p* > 0.10). Compared with the remote relatedness condition, there were also significantly higher hippocampal functional connectivity in the encoding of remembered trials in the “close” condition (*t*_(19)MFG_R_ = 2.70, *p* = 0.014; *t*_(19)IFG_R_ = 1.84, *p* = 0.081; *t*_(19)IPL_L_ = 3.42, *p* = 0.003; Figure 4). Notably, increased hippocampal functional coupling with the right inferior frontal gyrus was predictive of greater memory scores in the “close” condition (*r*_(predicted, observed)_ = 0.351, *p* = 0.041), but not in the “remote” condition (Figure 5).

#### 3.3.3 Trial‐by‐Trial Representational Similarity

**FIGURE 6:**
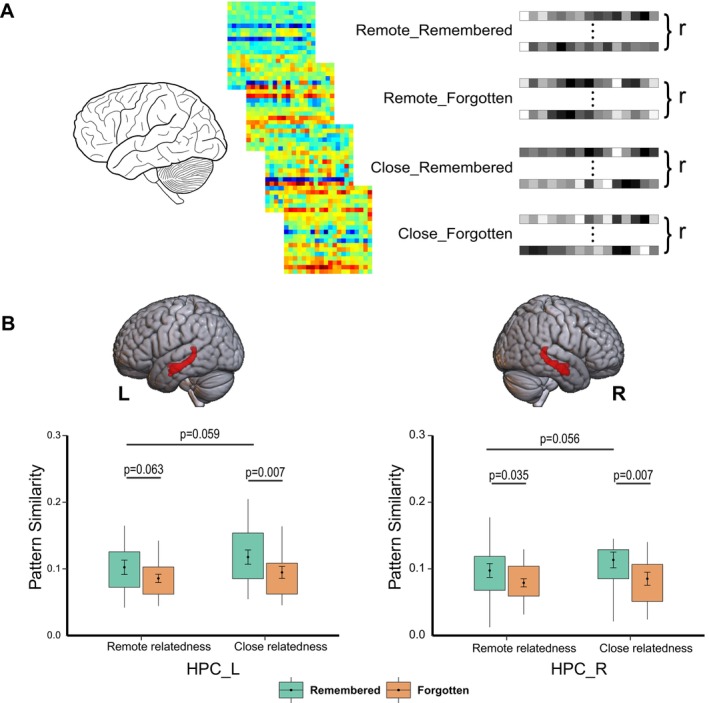
(A) Schematic depiction of inter‐item multivoxel pattern similarity analysis. The activation patterns were extracted from the defined ROIs for each item within the same condition and subjected to pairwise correlation analysis. The pattern similarity was calculated by averaging the Fisher's *z*‐scores of correlation coefficients. (B) Increased pattern similarity in the left and right hippocampus for remembered items in both two semantic relatedness conditions compared with forgotten items, and higher hippocampal representational similarity for the remembered items in the close relatedness condition compared with the remote condition. Box plots represent multivoxel pattern similarity in the left and right hippocampus. HPC, hippocampus; L, left; R, right.

To identify hippocampal neural pattern similarity associated with the encoding of creative associations in the “close” and “remote” conditions, we examined inter‐item correlational similarity of multivoxel activity patterns within each condition. This analysis revealed significant main effects of memory (*F*_(1, 19)HPC_L_ = 10.88, *p* = 0.004; *F*_(1, 19)HPC_R_ = 11.75, *p* = 0.003) and semantic relatedness (*F*_(1, 19)HPC_L_ = 5.50, *p* = 0.030; *F*_(1, 19)HPC_R_ = 4.83, *p* = 0.040) in the anatomically defined hippocampus, with greater multivoxel pattern similarity for remembered relative to forgotten items in both “close” (*t*_(19)HPC_L_ = 3.01, *p* = 0.007; *t*_(19)HPC_R_ = 3.02, *p* = 0.007) and “remote” conditions (*t*_(19)HPC_L_ = 1.97, *p* = 0.063; *t*_(19)HPC_R_ = 2.27, *p* = 0.035). Further analysis revealed a non‐significant trend of higher hippocampal representational similarity for remembered items in the close relatedness compared with the remote relatedness condition (*t*_(19)HPC_L_ = 2.00, *p* = 0.059; *t*_(19)HPC_R_ = 2.03, *p* = 0.056; Figure 6B). While for forgotten items, there was no significant difference between the two relatedness conditions (*t*_(19)HPC_L_ = 1.23, *p* = 0.232; *t*_(19)HPC_R_ = 0.88, *p* = 0.392).

## 4 Discussion

The present study investigated hippocampal processing during memory encoding of the creative object–AU combinations with remote or close semantic relatedness. The results revealed diverse patterns of hippocampal activity that predicted subsequent memory retrieval. On the one hand, multivariate analysis showed that during encoding, successfully retrieved trials exhibited greater inter‐item pattern similarity within the hippocampus in both the close and remote relatedness conditions. This suggests that irrespective of semantic relatedness, similar or consistent hippocampal multivoxel activity patterns across different trials support the successful encoding of creative associations, that is, such encoding relies more on integration processes than on purely item‐specific processing (Staresina and Davachi 2009; van Kesteren et al. 2012; Davis et al. 2014). On the other hand, univariate analyses revealed that hippocampal activation and its functional connectivity with prefrontal and parietal cortices differentially supported the successful encoding of creative associations with remote or close inherent semantic relatedness. While enhanced hippocampal activation during encoding was predictive of subsequent memory retrieval specifically in the “remote” condition, increased hippocampal connectivity with prefrontal and parietal cortices predicted memory retrieval in the “close” condition. These findings suggest that successful memory encoding of creative associations between semantically distant concepts may be a hippocampal‐dependent process; when the semantic distance of the to‐be‐associated elements is relatively close, successful memory for creative associations depends not on the hippocampus alone but rather on hippocampal functional coupling with prefrontal and parietal cortices, indicating a distributed hippocampal network pattern.

The finding that greater hippocampal engagement during encoding was associated with successful memory retrieval in the “remote” but not “close” condition does not fully align with previous findings on SME, in which enhanced hippocampal engagement consistently predicts subsequent mnemonic success (Prince et al. 2005; Davachi 2006; Uncapher et al. 2006; Eichenbaum et al. 2007; Chua et al. 2007; Kim 2011). In fact, we found that the “the stronger, the better” effects hold true only when the inherent semantic relatedness between the two to‐be‐combined elements (i.e., the object and its alternate use) is relatively remote. Under this circumstance, the hippocampus may contribute to the acquisition of a coherent representation, where two elements with relatively remote or weak semantic connections are bound together into a unified representation, thereby supporting cued memory retrieval (O'Reilly and Rudy 2001; Ranganath 2010; Zeithamova and Preston 2010; Zeithamova, Dominick, and Preston 2012; Horner et al. 2015; Schlichting and Preston 2015). In contrast, we found that in the “close” condition, enhanced hippocampal engagement during encoding predicted the forgetting rather than remembering of the encoded creative associations, which may be due to increased interference between relatively strong pre‐existing semantic connections and new creative associations whose encoding could be strengthened by greater hippocampal participation. Our results further revealed that the remembering of creative associations in the “close” condition was related to enhanced hippocampal coupling with the inferior frontal gyrus (IFG) and inferior parietal lobule (IPL), suggesting the involvement of a distributed hippocampal‐neocortical network during memory formation. Among the regions functionally coupled with the hippocampus, the role of IFG is often attributed to semantic processing, particularly involved in the controlled retrieval of semantic information to subject to current requirements (Thompson‐Schill et al. 1997; Wagner et al. 2001; Badre and Wagner 2007; Crescentini et al. 2010; Jefferies 2013; Krieger‐Redwood et al. 2023). Thus, enhanced functional connectivity between the hippocampus and IFG during successful memory encoding may imply more intensive semantic integration in the formation of creative associations. Specifically, in the “close” condition, successful memory encoding may be accompanied by an increased need for the retrieval and selection of the less dominant semantic information within element concepts, which facilitates the integrating of two concepts to form new creative associations. Meanwhile, increased hippocampal functional coupling with left IPL predicting subsequent memory retrieval suggests that there may also be an attention‐based integration process during successful memory encoding of creative associations. The IPL is thought to be part of the core network related to attentional processing and mental orientation in space, time, and person, supporting constructive simulation of new events (Schacter et al. 2007; Peer et al. 2015; Madore et al. 2016; Beaty et al. 2020). In the “close” condition, greater functional interaction between the left IPL and hippocampus may be associated with internally directed attention to the episodic representations during encoding. In sum, in the close relatedness condition, the successful encoding of creative associations could be achieved through a distributed hippocampal network rather than the hippocampus alone, implying associative processing modulated by semantic integration and attentional orientation.

From the perspective of hippocampal representation as revealed by pattern similarity analysis, in both the “close” and “remote” conditions, the inter‐item similarity of hippocampal activity patterns for remembered items was greater than that for forgotten items. The result suggests that more consistent or less separable hippocampal representations of encoded items may support successful memory formation. This may reflect the specific type of creative stimuli and memory test used in our study. Compared with other forms of memory retrieval, such as the recognition of a single item (e.g., a word or face), the cued‐recall test may require more cognitive effort to reconstruct encoded scenes, particularly in the case of creative associations. Greater inter‐item similarity of hippocampal activity patterns could contribute to forming such an integrated representation of encoded episodic scenes to facilitate subsequent memory retrieval. Moreover, compared with the remote relatedness condition, successfully retrieved items in the close relatedness condition exhibited a trend of higher inter‐item representational similarity in the hippocampus, suggesting that pre‐existing semantic connections may enhance hippocampal pattern integration supporting successful encoding.

The study investigated the neural mechanisms underlying the memory encoding of creative associations. Currently, only a few empirical studies and theoretical frameworks have focused on this issue, with one example being the prediction error theory (PE theory), which accounts for the memory advantage associated with insightful experiences (Becker and Cabeza 2025; Becker et al. 2025). According to the PE theory, prediction errors elicited during insight are detected by the hippocampus, and through hippocampal interactions with the medial prefrontal cortex, these insight events are preferentially consolidated into long‐term memory. While the theory is proposed in the context of creative insights, it may also extend to other forms of creative thinking. In line with this framework, our study demonstrates that the hippocampus and its associated network contribute to memory formation for creative associations. Although the present design lacked a non‐creative control condition, which may have constrained the direct examination of the memory advantage for creative associations and their neural correlates, participants provided subjective creativity assessments for each object‐AU combination during encoding. The assessment, to some extent, could serve as an indicator of prediction error, with items rated as creative assumed to elicit greater prediction errors, thereby allowing us to test the PE theory. According to the theory, there should be better memory for object‐AU combinations rated as creative, given their presumed generation of greater prediction errors. However, our results did not reveal such an advantage for object‐AU associations rated as creative relative to non‐creative ones, and there were no differences in either the remote or close relatedness conditions. This result appears inconsistent with the PE theory. One possible explanation is that the stimuli used in the present study were uniformly highly creative; thus, although participants subjectively perceived some items as creative or non‐creative during encoding, the actual differences may not have been sufficient to yield significant effects. These results suggest that a memory advantage for creative ideas is likely to emerge in cases where creativity levels differ markedly, as illustrated by the contrast between insight and non‐insight problem solving. In sum, our study demonstrates that distinct engagement of the hippocampus and its associated network supports the memory encoding of creative associations with remote or close inherent semantic relatedness, under conditions in which creativity (and possibly prediction error) is relatively well controlled.

Our findings provide new implications for understanding the neurocognitive basis of creative thinking, particularly highlighting the pivotal role of the hippocampus (Cabeza et al. 2020). Previous studies have established a widely accepted neurocognitive model suggesting that creative thinking is supported by coordinated functional interactions between the executive control network and default mode network (Beaty et al. 2016; Rabinovich et al. 2025). Specifically, the default network mediates bottom‐up, free‐associative thought, whereas the executive control network is involved in top‐down, controlled processing (Wei et al. 2014; Beaty et al. 2015; Shofty et al. 2022; Li et al. 2025; Luchini et al. 2025). These two processes are complementary in the generation of creative ideas, fostering cognitive searches that are simultaneously flexible, original, and goal‐directed. Critically, the hippocampus may function as a hub for large‐scale neural network interactions. As an important component of the default network, the hippocampus supports spontaneous associative thinking (including the bizarre associations characteristic of dreams) in the absence of explicit top‐down monitoring. It also contributes to the processing of novelty and its integration with usefulness under intentional cognitive control (Ren et al. 2023; Becker et al. 2025). One way the hippocampus achieves such multifaceted functions is through altering its interactions with other neural networks. A key finding of the present study is that the manner in which the hippocampus performs its basic associative function partly depends on the strength of pre‐existing semantic connections on which the creative associations are built. When the old associations are relatively weak, the hippocampus primarily plays a role on its own. In contrast, when old associations are relatively strong, reduced hippocampal involvement accompanied by increased hippocampal coupling with prefrontal and parietal cortices may be optimal, as it minimizes interference from old associations and facilitates the integration of new creative associations into existing semantic context.

Moreover, our results align with previous evidence indicating that the hippocampus and its network are selectively engaged in the encoding of new information which is incongruent or congruent with prior knowledge or schemas (Poppenk et al. 2010; van Kesteren et al. 2013; Preston and Eichenbaum 2013; Reggev et al. 2016; Liu et al. 2017, 2018). Our observations suggest that hippocampal‐specific associative processing alone supports memory for creative associations formed between elements with relatively remote semantic connections. This may represent a fundamental form of associative processing that is independent of pre‐existing knowledge and therefore less modulated by other functional networks. Hippocampal interactions with the prefrontal and parietal cortices, however, contribute to successful memory for creative associations which are formed between the elements with close semantic connections, suggesting that the hippocampus is more susceptible to top‐down modulation by the frontal–parietal network to enable integrative encoding. The findings may reflect that the degree of integration with pre‐existing knowledge influences hippocampal mechanisms: when integration demands are low, memory relies on hippocampal associative processing; when integration demands are high, hippocampal interactions with the frontal–parietal network are engaged. The present study did not detect activation in typical default network regions such as the posterior cingulate cortex (Shofty et al. 2022; Luchini et al. 2025), which may be attribute to the nature of our creative task, requiring participants to passively process experimenter‐provided creative combinations rather than actively generate novel ideas. Future studies could adopt creative idea generation tasks to directly examine the hub role of the hippocampus and its functional switching during interactions between the executive control and default networks.

A potential limitation of the present design is that it cannot precisely determine the extent to which participants' responses in the cued‐recall test were based on newly learned creative associations or on pre‐existing semantic connections between the two elements. However, two lines of evidence indicate that memory retrieval of alternate uses primarily relied on the mental reinstatement of the encoded creative associations. First, participants' reports during the post‐experimental interview demonstrated that their recollective experiences were predominantly vivid recollections of creative ideas, suggesting that retrieval was largely grounded in the reconstruction of creative associations. Second, if pre‐existing semantic connections significantly shaped memory retrieval—as evidenced by the well‐documented advantage in recalling semantically related word pairs over unrelated pairs—then memory performance in the close relatedness condition should have exceeded that in the remote relatedness condition; however, this pattern was not observed, indicating that retrieval in the cued‐recall test was unlikely to be directly based on pre‐existing semantic connections between the elements.

Another important consideration is that the four conditions in the present study were classified according to each participant's subsequent behavioral performance in the cued‐recall test and semantic relatedness judgment task. This classification resulted in unequal numbers of trials across conditions for each participant (Table S2), and especially in certain conditions too few trials were available, necessitating the exclusion of several participants. To estimate the possible impact of participant exclusion on the robustness of our main findings, we conducted additional analyses with expanded sample sizes of 23, 25, and 29 participants, ensuring at least six, five, or one trial per condition for each participant. All of these additional analyses replicated our original findings regarding the dissociable roles of the hippocampus and its network in mediating memory formation for creative associations with remote or close semantic relatedness (Methods S1–S3; Tables S3–S8; Figures S3–S11). Furthermore, on the one hand, previous studies have consistently proposed that there should be a sufficient number of trials in each condition to be averaged to improve estimation efficiency and statistical power in fMRI studies (Burock et al. 1998; Friston et al. 1999, 2007; Liu 2004; Liu and Frank 2004; Luo and Knoblich 2007). On the other hand, the number of subjects (sample size) could also affect the statistical power (Mumford and Nichols 2008; Mumford 2012; Soares et al. 2016). Thus, in the present study, we selected an optimal trade‐off between the number of trials and the number of participants. This is the reason why our primary analyses were based on the subset of 20 participants whose trial numbers in each condition met the acceptable threshold (Mumford and Nichols 2008; Fink et al. 2009; Mumford 2012; Benedek et al. 2018).

In conclusion, the findings of this study offer new insight into hippocampal mechanisms underlying memory for creative associations with remote or close inherent semantic relatedness. Multivariate analysis showed higher inter‐item hippocampal representational similarity for remembered items in both the close and remote relatedness conditions, suggesting that a more consistent neural representation of the learned episodes contributes to successful memory retrieval. Univariate analyses revealed differential hippocampal processing in the encoding of creative associations with remote or close semantic relatedness. Specifically, enhanced hippocampal activation predicted successful memory for creative associations formed between semantically remote concepts, suggesting hippocampal‐specific encoding processes; while successful encoding of creative associations between semantically close concepts was characterized by reduced hippocampal activation but increased engagement of a distributed hippocampal network, that is, hippocampal functional coupling with the inferior frontal gyrus and the inferior parietal lobule, indicating associative processing modulated by semantic integration and attentional orientation. These findings provide new evidence for multifaceted and dissociable neural representations supporting successful memory encoding of creative associations in the context of the interactions between pre‐existing semantic connections and newly formed creative associations.

## Conflicts of Interest

The authors declare no conflicts of interest.

## Supporting information


**Data S1:** hbm70381‐sup‐0001‐Supinfo.pdf.

## Data Availability

The data that support the findings of this study are available from the corresponding author upon reasonable request.
