# Supplementary material for: How the New Interacts With the Old? Hippocampal Processing During Memory Encoding of Creative Associations With Remote or Close Inherent Semantic Relatedness
Source: Hum Brain Mapp. 2025 Oct 23;46(15):e70381. doi: 10.1002/hbm.70381 (PMC12547841; doi:10.1002/hbm.70381)
Supplement: Supplementary file 1 — Data S1: hbm70381‐sup‐0001‐Supinfo.pdf. [file HBM-46-e70381-s001.pdf]

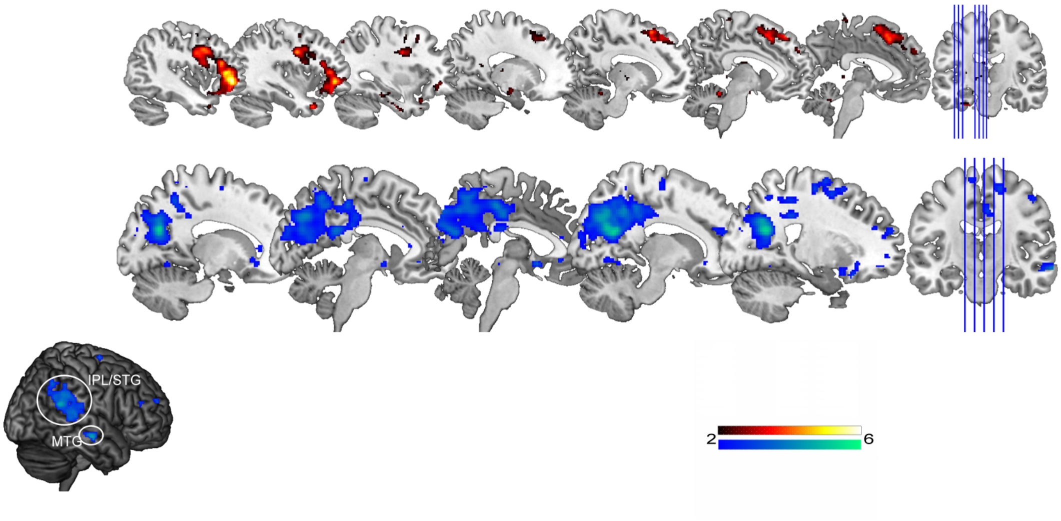

Figure S1 Brain regions associated with the main effect of memory. Brain activation associated with remembered > forgotten (in red) and forgotten > remembered (in blue). IPL, inferior parietal lobule; STG, superior temporal gyrus; MTG, middle temporal gyrus.

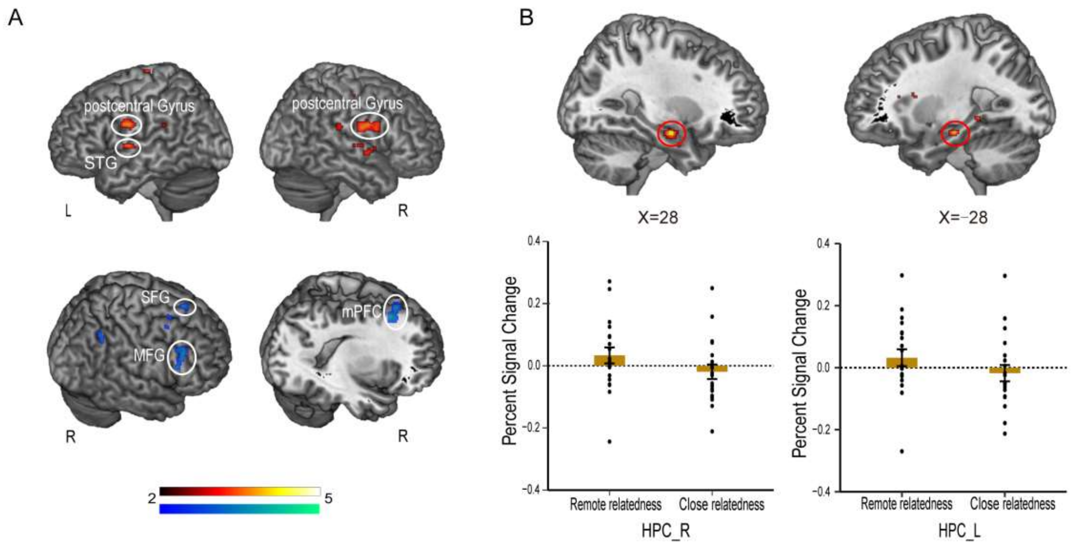

Figure S2 Brain regions associated with the main effect of semantic relatedness. (A) Brain activation associated with remote > close (in red) and close > remote (in blue). (B) Left (MNI, peak at -28, -20 and -16) and right hippocampus (MNI, peak at 28, -8 and -16) are involved in the remote relatedness condition in contrast to the close condition. Bar graphs represent functional activation in the left and right hippocampus showing higher engagement in the remote relative to the close relatedness condition. MNI, Montreal Neurological Institute; STG, superior temporal gyrus; SFG, superior frontal gyrus; MFG, middle frontal gyrus; mPFC, medial prefrontal cortex; HPC, hippocampus; R, right; L, left.

Table S1 Hippocampal functional connectivity changes underlying interaction effect.

Brain regions	Hemisphere	Brodmann's area	MNI Coordinates			*T*	K
			x	*y*	*z*		
**Interaction effect**							
Inferior Frontal Gyrus	right	47	48	36	-6	3.93	13
		45	58	26	20	3.67	17
Middle Frontal Gyrus	right	46	38	24	40	3.48	24
Putamen	right		26	14	-6	3.74	29
Inferior Parietal Lobule	left	7	-36	-60	54	3.69	16

Note: Threshold of voxel levels: T=3.20, p<.001 (uncorrected), K=10.

Table S2 The number of trials in scanning task.

Subjects	Remote_Remembered	Remote_Forgotten	Close_Remembered	Close_Forgotten
1	6	10	49	35
**2**	**33**	**15**	**41**	**11**
**3**	**13**	**35**	**12**	**40**
**4**	**27**	**35**	**13**	**25**
**5**	**17**	**16**	**34**	**33**
**6**	**16**	**30**	**27**	**27**
**7**	**13**	**44**	**12**	**31**
8	1	3	17	79
9	14	84	1	1
10	2	77	0	21
11	0	1	6	93
**12**	**27**	**12**	**43**	**18**
13	78	18	3	1
**14**	**10**	**23**	**28**	**39**
**15**	**39**	**24**	**29**	**8**
**16**	**43**	**38**	**10**	**9**
17	2	12	4	82
**18**	**22**	**50**	**9**	**19**
**19**	**14**	**13**	**38**	**35**
**20**	**26**	**25**	**23**	**26**
21	0	48	0	52
22	10	63	6	21
**23**	**41**	**25**	**19**	**15**
**24**	**25**	**48**	**8**	**19**
25	42	57	0	1
**26**	**30**	**42**	**8**	**20**
**27**	**8**	**39**	**13**	**40**
**28**	**10**	**10**	**45**	**35**
**29**	**44**	**17**	**27**	**12**
30	5	25	20	50
31	19	74	0	7
32	19	5	52	24
33	39	47	8	6
34	34	34	19	13

Note: Participants whose trial numbers in each conditions were at least eight and included in the formal statistical analysis are in bold.

Method S1:

In the current study, we collected data from 34 participants, and five were excluded for having no remembered items in either the remote or close relatedness conditions (Table S2). For the remaining 29 participants who had at least one trial in each condition, a 2-by-2 ANOVA showed significant interaction effects between semantic relatedness and memory in the right hippocampus and parahippocampal gyrus in the univariate GLM analysis (Table S3). Further ROI analyses revealed higher activation in the parahippocampal gyrus for remembered relative to forgotten trials in the remote condition ($t_{(28)PHG\_R}$ =4.19, p <0.001). In the close condition, we observed significant decreased activation in the right hippocampus for remembered relative to forgotten trials ($t_{(28)HPC\_R}$ = -2.77, p = 0.010, Figure S3). Moreover, using the right hippocampus as a seed, functional connectivity analysis revealed significant interaction effect in the right inferior frontal gyrus (Table S4). Further analysis revealed significantly higher hippocampal functional coupling with inferior frontal gyrus for remembered relative to forgotten items in the close condition ($t_{(28)IFG\_R}$ = 2.74, p = 0.011). In the remote relatedness condition, there was no significant difference between the remembered and forgotten items ($t_{(28)IFG\_R}$ = -1.89, p > 0.06, Figure S4). And because we computed the inter-item correlational similarity of activity patterns within each condition, the data from three participants were further excluded from multivoxel pattern similarity analysis. These participants (subject 8, subject 9, subject 13) had only one trial in one or two conditions, and the similarity of activity patterns between items could not be calculated. Then, the analysis showed a significant main effect of memory in the anatomically defined hippocampus ($F_{(1, 25)HPC\_L}$ = 5.89, p = 0.023; $F_{(1, 25)HPC\_R}$ = 6.10, p = 0.021), with higher hippocampal pattern similarity for remembered trials relative to forgotten trials in the close condition ($t_{(25)HPC\_L}$ = 3.63, p = 0.001; $t_{(25)HPC\_R}$ = 2.25, p = 0.034). Further analysis revealed a non-significant trend of higher hippocampal representational similarity for remembered trials in the close relatedness condtion compared with the remote condition ($t_{(25)HPC\_L}$= 2.01, p = 0.055, Figure S5).

Table S3 Brain regions associated with the interaction between semantic relatedness and memory (N=29).

Brain regions	Hemisphere	Brodmann's area	MNI Coordinates			T	K
			x	y	z		
**Interaction effect**							
Hippocampus	right		40	-12	-16	3.09	10
Parahippocampal Gyrus	right		36	-20	-20	3.32	9

Note: Threshold of voxel levels: T=2.62, p<0.005 (uncorrected).

Table S4 Hippocampal functional connectivity changes underlying interaction effect (N=29).

Brain regions	Hemisphere	Brodmann's area	MNI Coordinates			*T*	K
			x	*y*	*z*		
**Interaction effect**							
Inferior Frontal Gyrus	right	47	44	22	-8	3.65	16

Note: Threshold of voxel levels: T=3.16, p<.001 (uncorrected).

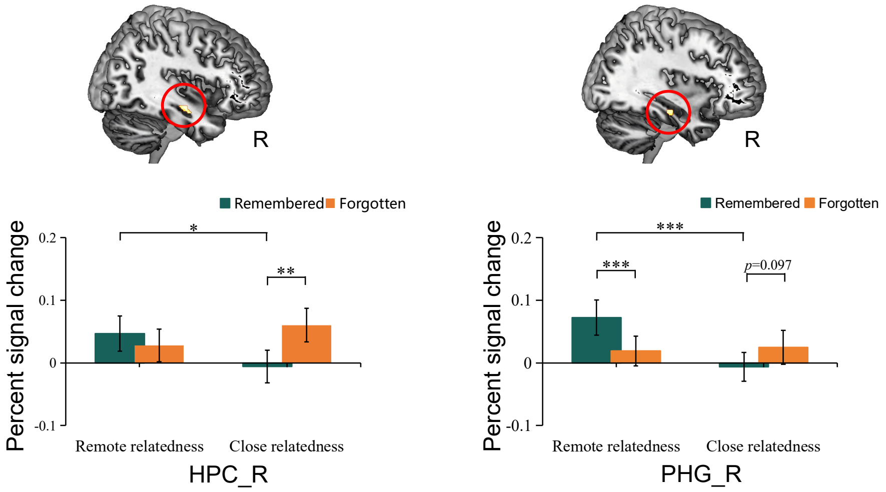

Figure S3 Significant clusters in the right hippocampus and parahippocampal gyrus showing interaction effects between semantic relatedness and memory (N=29). Bar graphs show that the percent signal changes in the ROIs which were obtained by superimposing anatomically defined masks on the functionally activated clusters in the interaction effects. *p<0.05; **p<0.01; ***p<0.001; HPC, hippocampus; PHG, parahippocampal gyrus; R, right.

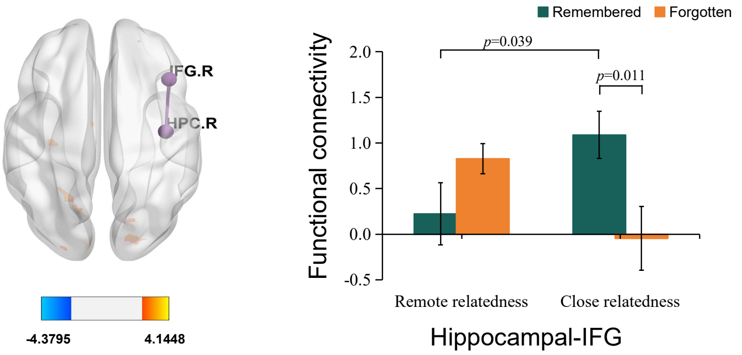

Figure S4 Significant clusters in the right IFG showing interaction effect between semantic relatedness and memory in gPPI functional connectivity analysis (N=29). Bar graphs represent hippocampal connectivity with right IFG for remembered items and forgotten items in the remote and close relatedness conditions. HPC, hippocampus; IFG, inferior frontal gyrus; R, right.

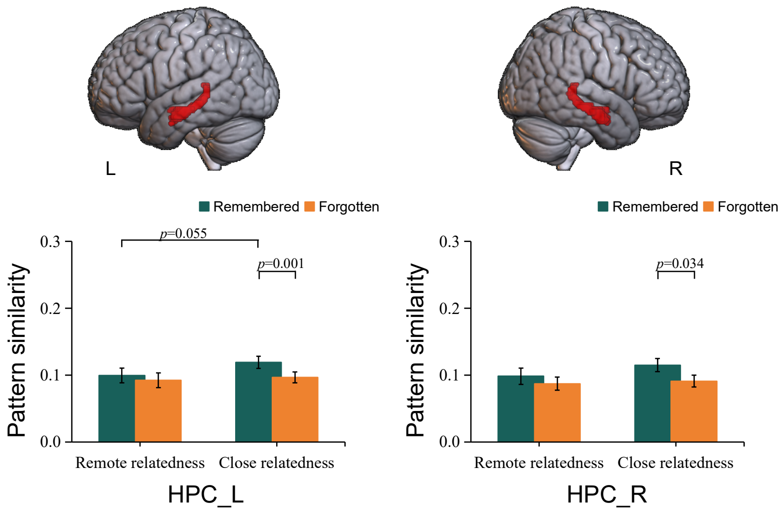

Figure S5 Increased pattern similarity in the left and right hippocampus for remembered items in the close relatedness condition compared with forgotten items, and higher hippocampal representational similarity for remembered items in the close relatedness condition compared with the remote condition (N=26). Box plots represent multivoxel pattern similarity in the left and right hippocampus. HPC, hippocampus; L, left; R, right.

Method S2:

For the 25 participants who at least five trials in each condition (Table S2), in the univariate GLM analysis, a 2-by-2 ANOVA showed significant interaction effects between semantic relatedness and memory in the bilateral hippocampus (Table S5). Further ROI analyses revealed higher activation in the bilateral hippocampus for remembered relative to forgotten trials in the remote condition ($t_{(24)HPC\_L} = 3.52$, $p = 0.002$; $t_{(24)HPC\_R} = 1.81$, $p = 0.083$). In the close condition, we observed significant decreased activation in the right hippocampus for remembered relative to forgotten trials ($t_{(24)HPC\_R} = -3.15$, $p = 0.004$, Figure S6). Moreover, using the bilateral hippocampus as seed regions, functional connectivity analysis revealed significant interaction effects in the middle frontal gyrus, inferior frontal gyrus, and inferior parietal lobule (Table S6). Further analyses revealed significantly higher hippocampal functional coupling with these regions for remembered relative to forgotten items in the close condition ($t_{(24)MFG\_R} = 2.64$, $p = 0.014$; $t_{(24)IFG\_R} = 2.75$, $p = 0.011$; $t_{(24)IPL\_L} = 2.90$, $p = 0.008$). In the remote relatedness condition, there was no significant difference between the remembered and forgotten items ($t_{(24)} < 1.93$, $p > 0.06$, Figure S7). Then, in the inter-item multivoxel pattern similarity analysis, the results showed a significant main effect of memory in the anatomically defined hippocampus ($F_{(1, 24)HPC\_L} = 6.03$, $p = 0.022$; $F_{(1, 24)HPC\_R} = 7.26$, $p = 0.013$), with higher hippocampal pattern similarity for remembered trials relative to forgotten trials in the close condition ($t_{(24)HPC\_L} = 3.43$, $p = 0.002$; $t_{(24)HPC\_R} = 2.14$, $p = 0.043$, Figure S8).

Table S5 Brain regions associated with the interaction between semantic relatedness and memory (N=25).

Brain regions	Hemisphere	Brodmann's area	MNI Coordinates			T	K
			x	y	z		
**Interaction effect**							
Hippocampus	right		40	-12	-16	3.30	18
Hippocampus	left		-30	-6	-16	3.37	17

Note: Threshold of voxel levels: T=2.63, $p<0.005$ (uncorrected).

Table S6 Hippocampal functional connectivity changes underlying interaction effect (N=25).

Brain regions	Hemisphere	Brodmann's area	MNI Coordinates			*T*	K
			x	*y*	*z*		
**Interaction effect**							
Inferior Frontal Gyrus	right	47	42	24	-18	3.41	32
		45	58	28	16	3.88	54
Middle Frontal Gyrus	right	46	34	26	38	3.15	21
Inferior Parietal Lobule	left	40	-58	-54	36	3.30	129

Note: Threshold of voxel levels: T=2.63, p<.005 (uncorrected).

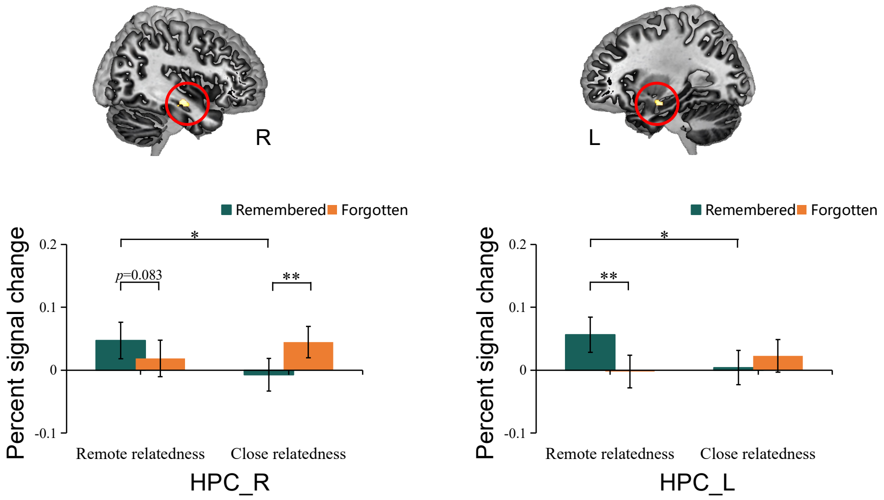

Figure S6 Significant clusters in the right and left hippocampus showing interaction effects between semantic relatedness and memory (N=25). Bar graphs show that the percent signal changes in the hippocampal ROIs which were obtained by superimposing anatomically defined masks on the functionally activated clusters in the interaction effects. *p<0.05; **p<0.01; HPC, hippocampus; L, left; R, right.

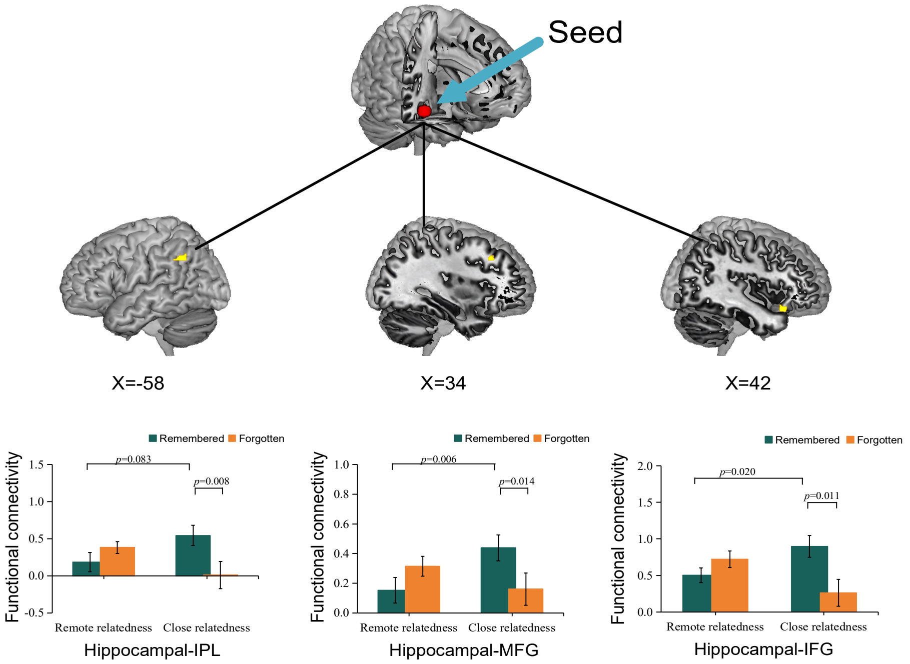

Figure S7 Significant clusters in the right IFG, MFG and left IPL showing interaction effects between semantic relatedness and memory in gPPI functional connectivity analysis (N=25). Bar graphs represent hippocampal connectivity with these regions for remembered items and forgotten items in the remote and close relatedness conditions. IFG, inferior frontal gyrus; MFG, middle frontal gyrus; IPL, inferior parietal lobule.

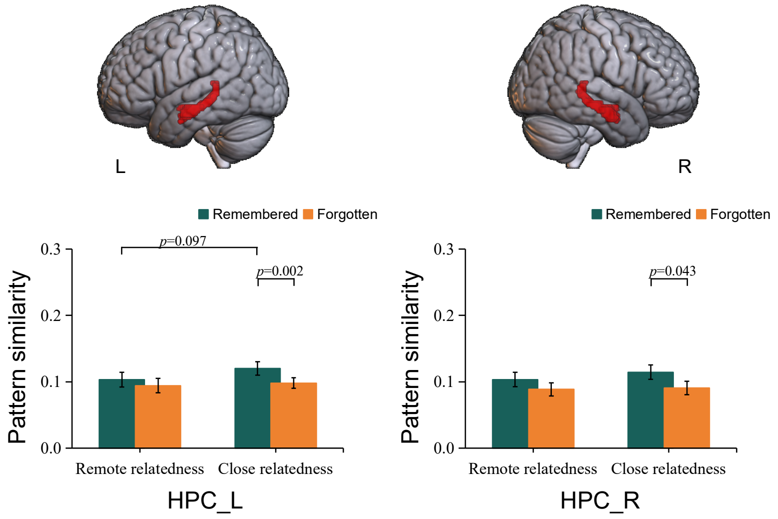

Figure S8 Increased pattern similarity in the left and right hippocampus for remembered items in the close relatedness condition compared with forgotten items, and higher hippocampal representational similarity for remembered items in the close condition compared with the remote relatedness condition (N=25). Box plots represent multivoxel pattern similarity in the left and right hippocampus. HPC, hippocampus; L, left; R, right.

Method S3:

For the 23 participants who at least six trials in each condition (Table S2), in the univariate GLM analysis, a 2-by-2 ANOVA showed significant interaction effects between semantic relatedness and memory in the bilateral hippocampus (Table S7). Further ROI analyses revealed higher activation in the bilateral hippocampus for remembered relative to forgotten trials in the remote condition ($t_{(22)HPC\_L}$ =3.20, *p* =0.004; $t_{(22)HPC\_R}$ = 2.90, *p* = 0.008). In the close condition, we observed significant decreased activation in the right hippocampus for remembered relative to forgotten trials ($t_{(22)HPC\_R}$ = -3.35, *p* = 0.003), and a similar but non-significant trend in the left hippocampus ($t_{(22)HPC\_L}$ =-1.67, *p* =0.109, Figure S9). Moreover, using the bilateral hippocampus as seed regions, functional connectivity analysis revealed significant interaction effects in the middle frontal gyrus, inferior frontal gyrus, and inferior parietal lobule (Table S8). Further analyses revealed significantly higher hippocampal functional coupling with these regions for remembered relative to forgotten items in the close condition ($t_{(22MFG\_R}$ =2.62, *p*=0.016; $t_{(22)IFG\_R}$ = 2.77, *p* = 0.011; $t_{(22)IPL\_L}$ = 3.24, *p* = 0.004). In the remote relatedness condition, there was no significant difference between the remembered and forgotten items ($t_{(22)}$ < 1.81, *p* > 0.08, Figure S10). Then, in the inter-item multivoxel pattern similarity analysis, the results showed significant main effects of memory ($F_{(1, 22)HPC\_L}$ = 10.03, *p* = 0.004; $F_{(1, 22)HPC\_R}$ = 6.81, *p* = 0.016) and semantic relatedness ($F_{(1, 22)HPC\_L}$ = 5.73, *p* = 0.026; $F_{(1, 22)HPC\_R}$ = 5.77, *p* = 0.025) in the anatomically defined hippocampus, with higher hippocampal pattern similarity for remembered trials relative to forgotten trials in the close condition ($t_{(22)HPC\_L}$= 3.44, *p* = 0.002; $t_{(22)HPC\_R}$= 2.09, *p* = 0.049). Further analysis revealed significantly higher hippocampal representational similarity for remembered trials in the close condition compared with the remote relatedness condition ($t_{(22)HPC\_L}$= 2.46, *p* = 0.022, Figure S11).

Table S7 Brain regions associated with the interaction between semantic relatedness and memory (N=23).

Brain regions	Hemisphere	Brodmann's area	MNI Coordinates			T	K
			x	y	z		
**Interaction effect**							
Hippocampus	right		40	-12	-14	3.72	54
Hippocampus	left		-30	-6	-16	3.50	21

Note: Threshold of voxel levels: T=2.63, *p*<0.005 (uncorrected).

Table S8 Hippocampal functional connectivity changes underlying interaction effect (N=23).

Brain regions	Hemisphere	Brodmann's area	MNI Coordinates			*T*	K
			x	*y*	*z*		
**Interaction effect**							
Inferior Frontal Gyrus	right	47	46	22	-8	3.49	145
		45	58	28	14	4.10	65
Middle Frontal Gyrus	right	46	42	20	38	3.21	11
Inferior Parietal Lobule	left	40	-34	-70	50	3.65	102

Note: Threshold of voxel levels: T=2.63, *p*<.005 (uncorrected).

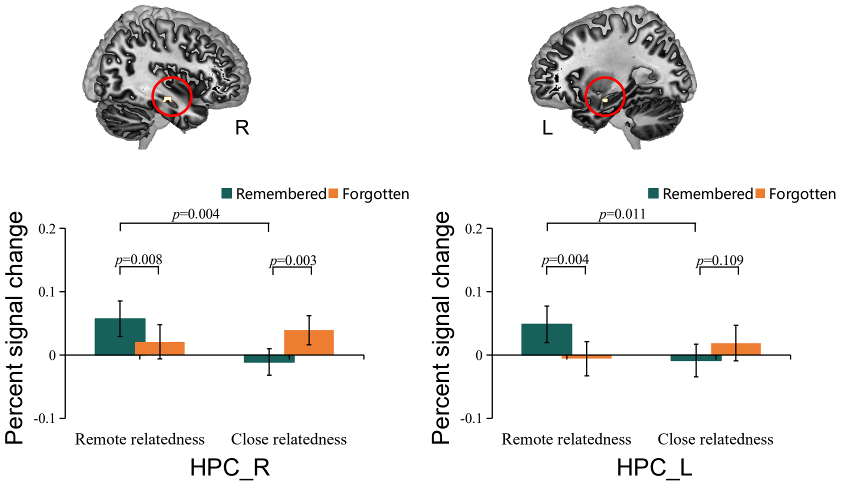

Figure S9 Significant clusters in the right and left hippocampus showing interaction effects between semantic relatedness and memory (N=23). Bar graphs show that the percent signal changes in the hippocampal ROIs which were obtained by superimposing anatomically defined masks on the functionally activated clusters in the interaction effects. HPC, hippocampus; L, left; R, right.

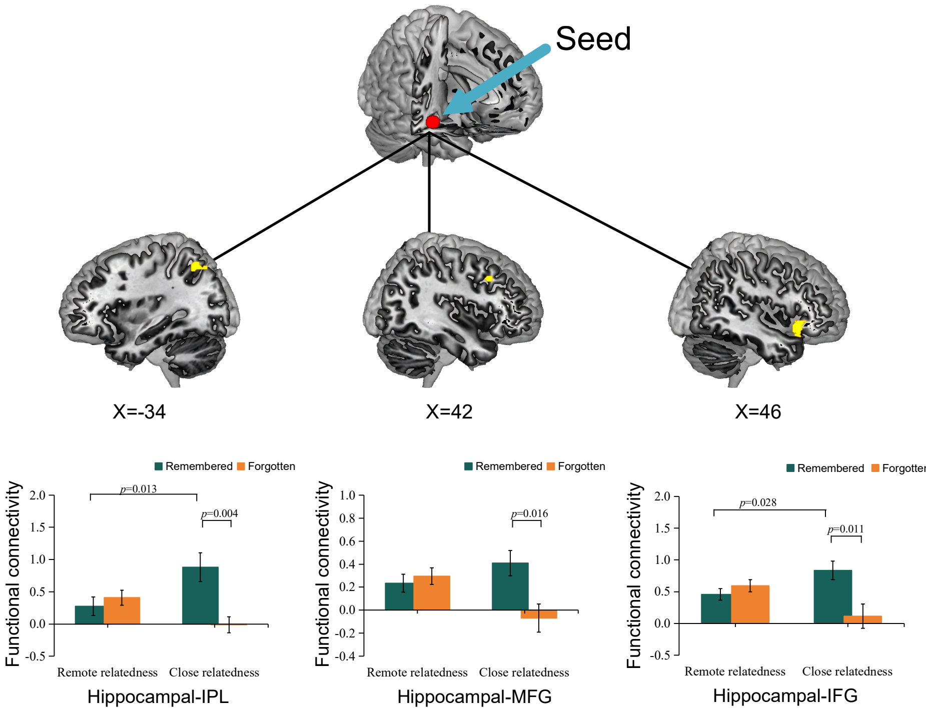

Figure S10 Significant clusters in the right IFG, MFG and left IPL showing interaction effects between semantic relatedness and memory in gPPI functional connectivity analysis (N=23). Bar graphs represent hippocampal connectivity with these regions for remembered items and forgotten items in the remote and close relatedness conditions. IFG, inferior frontal gyrus; MFG, middle frontal gyrus; IPL, inferior parietal lobule.

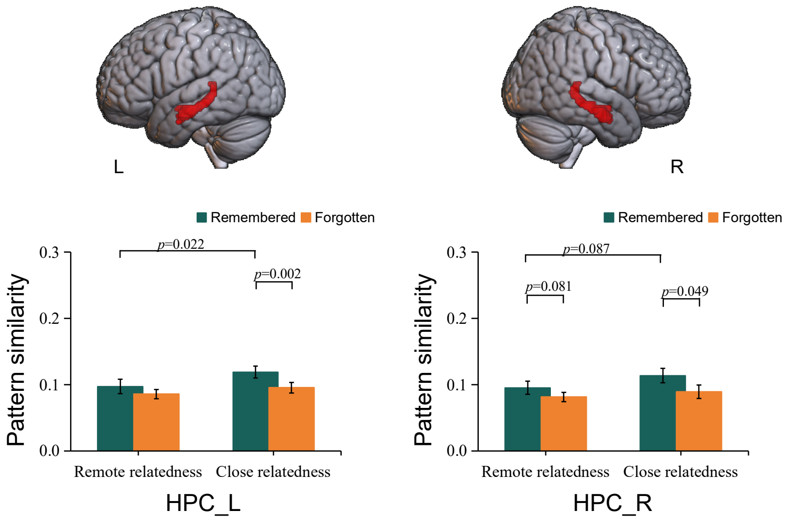

Figure S11 Increased pattern similarity in the left and right hippocampus for remembered items in the close relatedness condition compared with forgotten items, and higher hippocampal representational similarity for remembered items in the close condition compared with the remote relatedness condition (N=23). Box plots represent multivoxel pattern similarity in the left and right hippocampus. HPC, hippocampus; L, left; R, right.
